# In Silico Phosphoproteomic Analysis Reveals Divergent Regulation of Presenilin 1 and Presenilin 2

**DOI:** 10.1007/s12017-026-08906-z

**Published:** 2026-01-30

**Authors:** Sadia Begum, Javier Andres De Alvarez, Claudia Manzoni, Charlie Arber, Patrick A. Lewis

**Affiliations:** 1 Kings College London, Great Maze Pond, London, SE1 1UL UK; 2 Royal Veterinary College, Camden Town, NW1 0TU UK; 3 UCL School of Pharmacy, Brunswick Square, WC1N 1AX London, UK; 4 UCL Queen Square Institute of Neurology, Queen Square, London, WC1N 3BG UK

**Keywords:** Presenilin, Gamma secretase, Signal transduction, Alzheimer’s disease

## Abstract

The Presenilins are multi-pass transmembrane proteins that form part of the multi-protein gamma secretase complex. The hydrolytic activity of the gamma secretase complex is responsible for the cleavage of a wide range of substrates, including the amyloid precursor protein (APP) – a proteolytic event that is the final step in the production of the amyloid beta peptide, a protein fragment deposited in the brains of individuals with Alzheimer’s disease (AD). Both *PSEN1* and *PSEN2*, the genes encoding the Presenilins, are mutated in familial AD, generating intense interest in the activity and function of these proteins. Despite this attention, the post-translational modification and regulation of the Presenilins is poorly understood. In order to address this gap in our knowledge, a bioinformatic approach was taken to examine the extant evidence for Presenilin phosphorylation. Derived from the Phosphosite repository, these data reveal divergent patterns of phosphorylation across Presenilin 1 and 2, highlighting distinct regulatory pathways that have implications for our understanding of the biology of these proteins, gamma secretase, and drug discovery targeting this complex.

**Supplementary Information:**

The online version contains supplementary material available at 10.1007/s12017-026-08906-z.

## Introduction

Alzheimer’s disease (AD) is the leading cause of dementia, accounting for between 60 and 80% of cases worldwide, and is characterised by progressive cognitive decline and deficits in memory and reasoning (Scheltens et al., 2021). This is driven by neuronal loss, a consequence of cellular dysfunction associated with the accumulation of extracellular amyloid beta protein as amyloid plaques, and tau protein as intracellular neurofibrillary tangles.

The majority of AD cases are idiopathic, typically occurring in individuals over the age of 65, and influenced by a combination of age-related factors and genetic risk (Mayeux & Stern, 2012). A small proportion, estimated to be around 1%, of AD cases are familial, resulting from mutations inherited in an autosomal Mendelian fashion (Andrews et al., 2023). A key insight from studying genetic forms of AD has been the identification of the production of the amyloid beta peptide as a central event in the disease process (Tcw & Goate, 2017). Coding mutations in the *APP* gene on chromosome 21, encoding the amyloid precursor protein (APP), are causative for AD, as are copy number variants (including gene duplications and trisomy 21). APP, a type I transmembrane protein, is subject to multiple proteolytic processing events, with sequential cleavage by beta and gamma secretase activities producing the amyloid beta peptide. Importantly, disease associated gene variants in APP result in either an increase in amyloid beta production, or the generation of peptides with a greater propensity to aggregate. Two further genes linked to familial forms of AD, *PSEN1* on chromosome 14 and *PSEN2* on chromosome 1, contribute to the generation of amyloid beta. Presenilin 1 and Presenilin 2, the multi-pass integral membrane proteins encoded by *PSEN1* and *PSEN2* respectively, are close paralogs and essential components of the multi-protein gamma secretase proteolytic complex – contributing catalytic residues to the active site responsible for cleaving APP (Wolfe, 2020). Coding mutations in these genes result in altered processing of APP, favouring the production of longer (and more aggregation prone) peptides. Due to its central role in the etiopathogenesis of AD, the production of amyloid beta peptides has been a priority drug target for this disorder for several decades, with inhibitors of both gamma and beta secretase progressing to advanced clinical trials. To date, however, these trials have not demonstrated any clinical benefits, in contrast to efforts targeting the removal of amyloid beta by passive immunotherapy. Part of the reason for this may be the multi-protein nature of gamma secretase, incorporating Nicastrin, PEN-2 and Aph1a or Aph1b as well as Presenilin 1 or Presenilin 2, coupled to its wide range of proteolytic substrates, make it a challenging target for direct modulation (Wolfe, 2020; Guner & Lichtenthaler, 2020). Notably, there is evidence that the Presenilins can be regulated by phosphorylation, with signal transduction pathways governing their stability and activity (Ledo et al., 2021; Lau et al., 2002). In this current study, an in silico bioinformatic approach was taken to assess the post-translational modification of the Presenilins by phosphorylation, with the goal of characterising and understanding the physiological scale and scope of phospho-regulation of these proteins.

## Methods

To examine the extant evidence for phosphoregulation of the Presenilins, the post-transcriptional modification compendium web portal Phosphosite (https://www.phosphosite.org/) was accessed using PSEN1 and PSEN2 as the search terms (Hornbeck et al., 2015). (https://www.phosphosite.org/proteinAction.action?id=1633&showAllSites=true, https://www.phosphosite.org/proteinAction.action?id=1648&showAllSites=true). To assess whether any of these modifications are conserved across the two paralogs, the phosphorylation sites reported on Phosphosite were then mapped onto a BLAST generated Needleman-Wunsch alignment of Presenilin 1 and 2.

## Results and Discussion

**Fig. 1:**
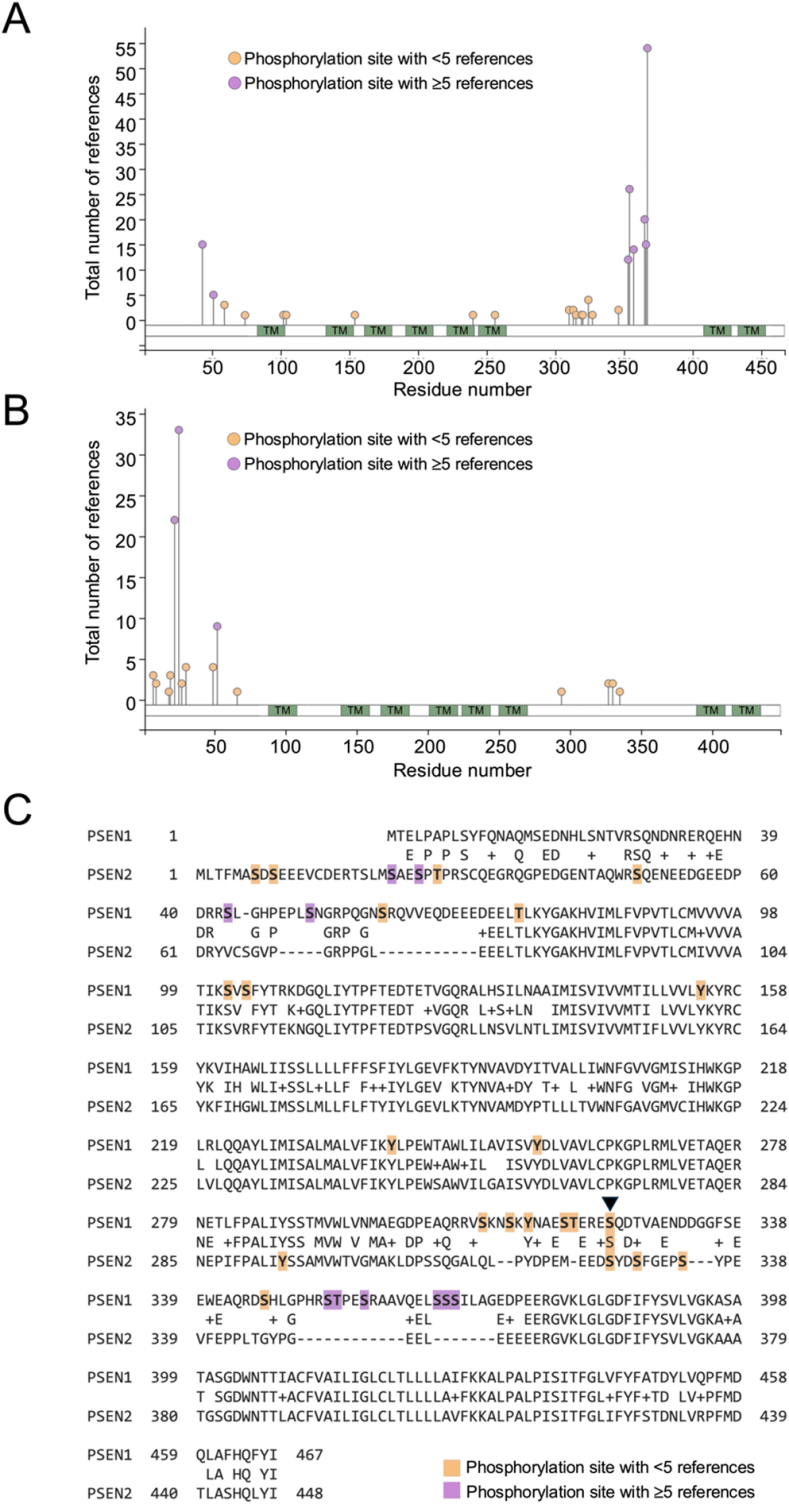
Phosphomodification of Presenilin 1 and 2 (**A**) Lollipop plot for Presenilin 1, showing reported phosphorylation events on serine, threonine and tyrosine residues. Orange lollipops represent events with fewer than 5 references, purple equal to or greater than 5 references. Transmembrane regions indicated by TM. (**B**) Lollipop plot for Presenilin 2, showing reported phosphorylation events on serine, threonine and tyrosine residues. Orange lollipops represent events with fewer than 5 references, purple equal to or greater than 5 references. Transmembrane regions indicated by TM. (**C**) Needleman-Wunsch sequence alignment for the primary amino acid sequence of Presenilin 1 and Presenilin 2, highlighting reported phosphorylation sites. Orange shading indicates events with fewer than 5 references, purple equal to or greater than 5 references. arrowhead indicates phosphorylation on residues conserved across Presenilin 1 and 2. Lollipop plots derived and modified from Phosphosite

The resulting data for canonical serine, threonine or tyrosine phosphorylation is presented as lollipop plots in Fig. 1A and B, with detailed information for each modification available in supplemental Tables 1 and 2 and *via* the Phosphosite portal. Strikingly, only one of the reported phosphorylation sites (residue S324 in Presenilin 1 and residue S327 in Presenilin 2) is conserved across both proteins, with all other sites being unique to one or other of the proteins (Fig. 1C). This divergence is consistent with the primary sequence organization of the proteins.

**Fig. 2:**
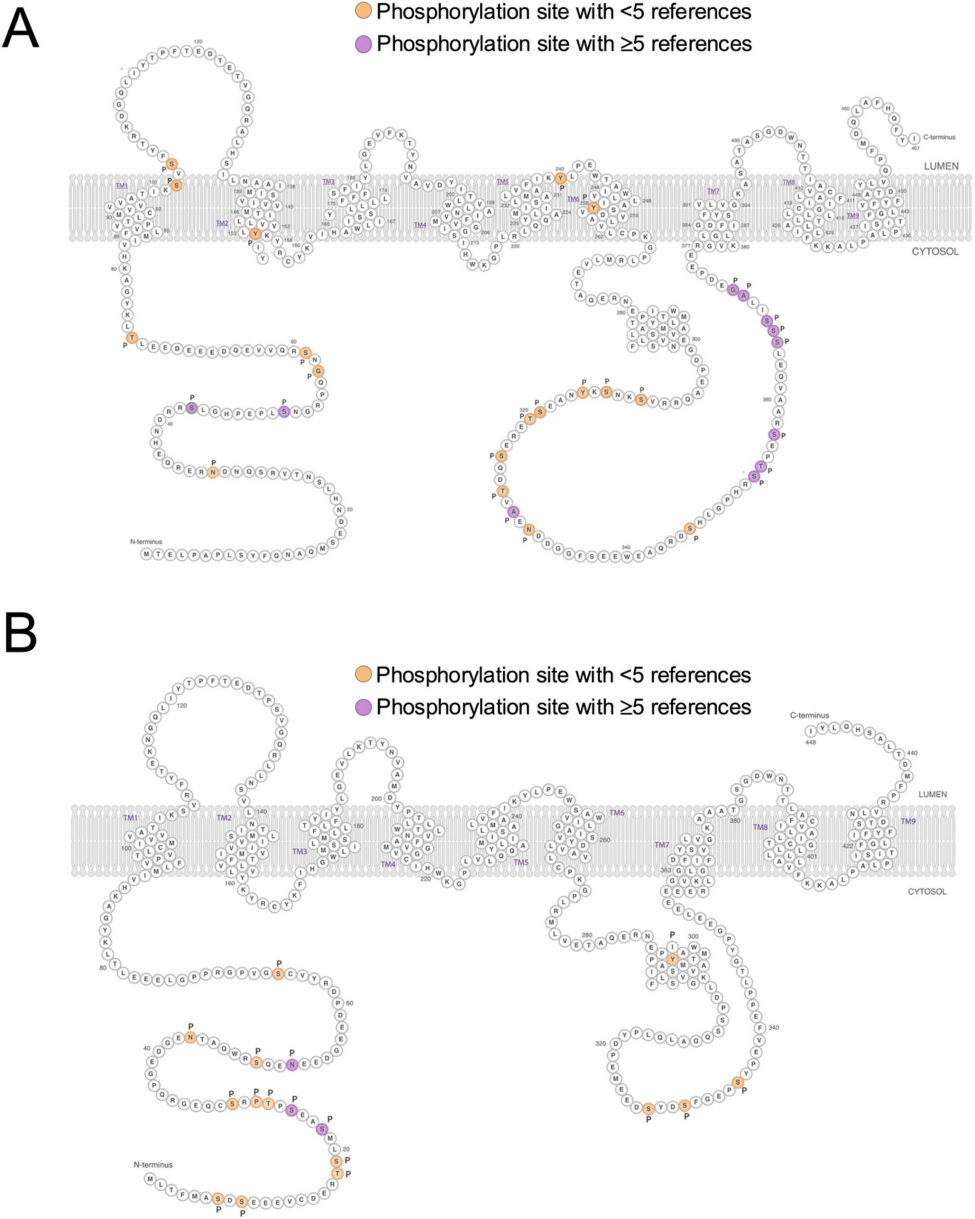
Ribbon diagram representing reported phosphorylation sites on the primary sequence of Presenilin 1 (**A**) and Presenilin 2 (**B**). Orange shading indicates amino acid phosphorylation event with fewer than 5 references, purple equal to or greater than 5 references. Ribbon diagrams modified from original images developed by Alzforum, reproduced with permission

To gain an insight as to the orientation of these residues within the Presenilins, the amino acids modified by phosphorylation were identified in simplified ribbon diagrams for Presenilin 1 and Presenilin 2 (Fig. 2). Although atomic resolution cryogenic electron microscopy (cryoEM) structures have been derived for gamma secretase containing both Presenilin 1 and Presenilin 2, it is of note that the residues catalogued in this study as being phosphorylated all sit in portions of their primary sequence that are not visible in these structures (PDB 5A63 and PDB 7Y5Z). This is likely due to their residing in flexible portions of the gamma secretase complex that are not amenable to visualisation by cryoEM.

The pattern of phosphorylation observed across Presenilin 1 and 2 is striking, and has some important implications. Most notably, and surprisingly, there is a clear divergence between the two Presenilins with regard to the location of phosphorylation sites. As noted above, Presenilin 1 and 2 are paralogs, sharing 63% identity and 7% similarity, and have a shared and redundant function - at least at a biochemical level - in the gamma secretase complex. This divergence is consistent with the primary sequence organization of the proteins. While Presenilin 1 and 2 are highly conserved within their transmembrane domains, many of the non-conserved phosphorylation sites map to cytosolic regions, specifically the N-terminal region and the large cytosolic loop. These regions exhibit reduced sequence conservation between Presenilin 1 and 2, likely contributing to the largely non-overlapping phosphorylation landscape by providing distinct kinase recognition motifs and regulatory interfaces in each paralog.

The human genetics of the *PSEN* genes indicates a closely aligned pathological role, with mutations in either gene resulting in a common phenotype. The observation that phosphoregulation of these proteins differs, however, suggests distinct roles within cells and tissues for each of the Presenilins, and implies that there are unique regulatory events governing these proteins. This opens up the possibility of specifically modulating the Presenilins individually *via* targeted inhibition and activation of upstream kinases – potentially indirectly modifying APP processing, and amyloid beta homeostasis through altered localisation, trafficking, or turnover of gamma secretase complexes, rather than direct changes in catalytic specificity.

Beyond the divergence in phosphorylation patterns, several studies have highlighted functional consequences of presenilin phosphorylation. Walter and co-workers reported that phosphorylation of Presenilin 2 near caspase cleavage sites reduces caspase-mediated proteolysis, delays apoptotic progression and protects Presenilin 2 from cleavage, whereas phospho-deficient Presenilin 2 is more readily cleaved (Walter et al., 1999). Similarly, Presenilin 1 phosphorylation has been shown to be critical for microglial phagolysosomal competence, including lysosomal acidification and efficient degradation of amyloid beta oligomers, effects that appear at least partly independent of canonical gamma secretase activity (Ledo et al., 2021). Mechanistic studies support these functional roles: for example, Presenilin 2 contains a phosphorylation-dependent AP-1 adaptor binding motif that directs Presenilin 2/gamma-secretase to late endososomes and lysosomes, a motif absent in Presenilin 1, explaining the preferential contribution of Presenilin 2 to APP processing in endolysosomal compartments (Sannerud et al., 2016). The observed sequence divergence in cytosolic loops, and the functional characterisation noted above, indicate that phosphorylation has been tailored to confer paralog-specific functional specialisation, regulating protein stability, trafficking stress responses and cell-type specific functions beyond classical gamma secretase cleavage activity.

There are a number of caveats to interpreting the phosphorylation events catalogued in this study. The underlying data represented on the Phosphosite portal includes both high-throughput unbiased analyses and low-throughput targeted analysis, however it is of note that only a minority of the phosphorylation sites across Presenilin 1 and 2 have been reported by more than five independent analyses. With decreased numbers of replication studies comes a decreased confidence in the physiological relevance of the phosphorylation event, a qualification that is relevant to the S324/S327 conserved phosphosites where there are 4 and 2 supporting references respectively. Several factors likely contribute to this limited overlap and validation: historical research bias, in which familial AD mutations in *PSEN* genes attracted the majority of attention toward studying mutational effects rather than post-translational regulation; technical challenges, since presenilins are low abundance, multipass membrane proteins that are difficult to solubilize and study by mass spectrometry; and context dependence, where phosphorylation events may be transient, cell-type-specific, activity-dependent, or stress-induced, limiting reproducibility across studies. The limited number of reports does not imply that these modifications are biologically irrelevant, as many phosphosites may exert context dependent effects on protein localisation, stability, or function. Consistent with this, Matz and co-workers identified 11 novel Presenilin 1 phosphosites but observed minimal effects on gamma secretase activity and abeta production under the tested conditions, suggesting that many of these phosphosites are permissive rather than universally instructive (Matz et al., 2015).

In addition, although Phosphosite compiles data on a wide range of post-translational modifications, encompassing acetylation, ubiquitylation and multiple classes of phosphorylation, this study has focused on reported canonical serine, threonine, and tyrosine phosphorylation events. This has the benefit of a wider evidence base for phosphorylation events, but has the drawback of missing the wider landscape of posttranslational modification as a means of regulating Presenilin function. A broader examination of posttranslational modification is beyond the scope of this current study, but is certainly merited in the future (especially in the light of rapidly increasing sensitivity and volume of proteomic analysis).

Finally, only a limited number of the phosphorylation events covered by this study have been characterised and validated in detail, with even fewer data available on the functional consequences of posttranslational regulation. Defining and understanding the signal transduction pathways that govern the phosphorylation of the Presenilins, and gaining a deeper comprehension of how these modifications alter Presenilin function, localization, stability, and interaction networks, should be prioritised in future investigations.

## Conclusion

This analysis represents the first comprehensive examination of phosphorylation patterns for Presenilin 1 and 2, revealing a surprising level of divergence between these two proteins. As core components of gamma secretase, and important contributors to genetic risk for AD, these data open new avenues for understanding Presenilin function as well as potential paths for drug discovery relevant to AD.

Phosphorylation of Presenilin 1 has been shown to orchestrate protein stability, and activity in pathways such as apoptosis and phagocytosis; together demonstrating the importance of post translational modification in modulating catalytic activity (Lau et al., 2002; Fluhrer et al., 2004; Ledo et al., 2021). Under specific signalling conditions, phosphorylation of Presenilin 1 at residues S365-367 has also been reported to induce a conformational state of gamma secretase associated with altered amyloid beta 42/40 ratios, suggesting a context-dependent influence of presenilin phosphorylation on amyloid processing rather than a general shift in catalytic specificity (Maesako et al., 2017), *id est* this could be explained by changes in enzyme to substrate complex interactions, rather than overt catalytic activity (Szaruga et al., 2017).

The two phospho-epitopes that are consistent between Presenilin 1 and Presenilin 2 (S324/S327, Fig. 1C) have both been implicated in apoptosis pathways, suggesting shared functional effects of parallel phosphorylation of the two presenilin paralogs (Wu et al., 2012; Walter et al., 1999). Conversely, the observed general divergence in phosphorylation patterns of Presenilin 1 and Presenilin 2 likely reflects specialisation rather than redundancy, pointing to distinct functional roles of Presenilin 1 and 2. This may help direct specificity of gamma secretase to its wide-ranging substrate repertoire, and be associated with the differing cellular and subcellular enrichment of the two proteins (Jayadev et al., 2010; Sannerud et al., 2016). Indeed, the substrate repertoire of Presenilin 1 was recently extended through a study of gamma secretase substrates in novel cell types (Guner & Lichtenthaler, 2020; Hou et al., 2023).

Collectively, these findings support a model in which Presenilin phosphorylation primarily regulates gamma-secretase localisation, trafficking and functional context, with more limited and context-dependent effects on catalytic cleavage specificity. These data reinforce the functional diversity of gamma secretase and provide evidence calling for the development of specific therapeutic targeting strategies (Luo & Li, 2022).

## Supplementary Information

Below is the link to the electronic supplementary material.

Supplementary material 1 (DOCX 30.7 kb)

## Data Availability

Data is available as supplemental material and trough the phosphosite plus web portal.
