# Supplementary material for: In Silico Phosphoproteomic Analysis Reveals Divergent Regulation of Presenilin 1 and Presenilin 2
Source: Neuromolecular Med. 2026 Jan 30;28(1):5. doi: 10.1007/s12017-026-08906-z (PMC12858466; doi:10.1007/s12017-026-08906-z)
Supplement: Supplementary file 1 — Supplementary material 1 (DOCX 30.7 kb) [file 12017_2026_8906_MOESM1_ESM.docx]

**Supplemental table 1** – Reported phosphorylated residues in the Presenilin 1 open reading frame, noting amino acid residue modified, the flanking epitope, and the number of references (high throughput, low throughput, and total). Data accessed and downloaded from the Phosphosite web portal.

**Supplemental table 2** - Reported phosphorylated residues in the Presenilin 2 open reading frame, noting amino acid residue modified, the flanking epitope, and the number of references (high throughput, low throughput, and total). Data accessed and downloaded from the Phosphosite web portal.

**Tables**

| Amino acid | Phosphorylated epitope | LTP | HTP | Total |
| --- | --- | --- | --- | --- |
| S43 | QEHNDRR**s**LGHPEPL | 0 | 15 | 15 |
| S51 | LGHPEPL**s**NGRPQGN | 0 | 5 | 5 |
| S59 | NGRPQGN**s**RQVVEQD | 0 | 3 | 3 |
| T74 | EEEDEEL**t**LkYGAKH | 1 | 0 | 1 |
| S102 | VVVATIK**s**VSFYTRK | 0 | 1 | 1 |
| S104 | VATIKSV**s**FYTRKDG | 0 | 1 | 1 |
| Y154 | TILLVVL**y**KYRCYKV | 0 | 1 | 1 |
| Y240 | MALVFIK**y**LPEWTAW | 0 | 1 | 1 |
| Y256 | ILAVISV**y**DLVAVLC | 0 | 1 | 1 |
| S310 | PEAQRRV**s**KNSKYNA | 2 | 0 | 2 |
| S313 | QRRVSKN**s**KYNAEST | 1 | 1 | 2 |
| Y315 | RVSKNSK**y**NAESTER | 0 | 1 | 1 |
| S319 | NSKYNAE**s**TERESQD | 1 | 0 | 1 |
| T320 | SKYNAES**t**ERESQDT | 1 | 0 | 1 |
| S324 | AESTERE**s**QDTVAEN | 0 | 4 | 4 |
| S346 | EWEAQRD**s**HLGPHRS | 1 | 1 | 2 |
| S353 | SHLGPHR**s**TPESRAA | 3 | 9 | 12 |
| T354 | HLGPHRS**t**PESRAAV | 2 | 24 | 26 |
| S357 | PHRSTPE**s**RAAVQEL | 3 | 11 | 14 |
| S365 | RAAVQEL**s**SSILAGE | 2 | 18 | 20 |
| S366 | AAVQELS**s**SILAGED | 1 | 14 | 15 |
| S367 | AVQELSS**s**ILAGEDP | 3 | 51 | 54 |

Supplemental table 1 – Presenilin 1

| Amino acid | Phosphorylated epitope | LTP | HTP | Total |
| --- | --- | --- | --- | --- |
| S7 | -MLTFMA**s**DSEEEVC | 3 | 0 | 3 |
| S9 | LTFMASD**s**EEEVCDE | 2 | 0 | 2 |
| S19 | EVCDERT**s**LMSAESP | 2 | 1 | 3 |
| S22 | DERTSLM**s**AESPTPR | 0 | 22 | 22 |
| S25 | TSLMSAE**s**PTPRSCQ | 0 | 33 | 33 |
| T27 | LMSAESP**t**PRSCQEG | 0 | 2 | 2 |
| S49 | ENTAQWR**s**QENEEDG | 0 | 4 | 4 |
| Y294 | PIFPALI**y**SSAMVWT | 0 | 1 | 1 |
| S327 | DPEMEED**s**YDSFGEP | 2 | 0 | 2 |
| S330 | MEEDSYD**s**FGEPSYP | 2 | 0 | 2 |
| S335 | YDSFGEP**s**YPEVFEP | 1 | 0 | 1 |

Supplemental table 2 – Presenilin 2
